# Effects of comorbid alcohol use disorder on the clinical outcomes of first-episode schizophrenia: a nationwide population-based study

**DOI:** 10.1186/s12991-021-00353-3

**Published:** 2021-05-29

**Authors:** Soojin Ahn, Youngjae Choi, Woohyeok Choi, Young Tak Jo, Harin Kim, Jungsun Lee, Sung Woo Joo

**Affiliations:** Department of Psychiatry, Asan Medical Center, University of Ulsan College of Medicine, 88 Olympic-Ro 43-Gil, SongPa-Gu, Seoul, 05505 Republic of Korea

**Keywords:** Schizophrenia, Alcohol use disorder, Comorbidity, Drug compliance, Hospitalization

## Abstract

**Background:**

Alcohol use disorder (AUD) is a common psychiatric comorbidity in schizophrenia, associated with poor clinical outcomes and medication noncompliance. Most previous studies on the effect of alcohol use in patients with schizophrenia had limitations of small sample size or a cross-sectional design. Therefore, we used a nationwide population database to investigate the impact of AUD on clinical outcomes of schizophrenia.

**Methods:**

Data from the Health Insurance Review Agency database in South Korea from January 1, 2007 to December 31, 2016 were used. Among 64,442 patients with first-episode schizophrenia, 1598 patients with comorbid AUD were selected based on the diagnostic code F10. We performed between- and within-group analyses to compare the rates of psychiatric admissions and emergency room (ER) visits, and medication possession ratio (MPR) between the patients with comorbid AUD and control patients matched for the onset age, sex, and observation period.

**Results:**

The rates of psychiatric admissions and ER visits in both groups decreased after the time point of diagnosis of AUD; however, the decrease was significantly greater in the patients with comorbid AUD compared to the control patients. While the comorbid AUD group showed an increase in MPR after the diagnosis of AUD, MPR decreased in the control group. The rates of psychiatric admissions, ER visits, and MPR were worse in the comorbid AUD group both before and after the diagnosis of AUD.

**Conclusions:**

The results emphasize an importance of psychiatric comorbidities, especially AUD, in first-episode schizophrenia and the necessity of further research for confirmative findings of the association of AUD with clinical outcomes of schizophrenia.

## Introduction

Alcohol use disorder (AUD) is a common psychiatric comorbidity in schizophrenia. According to a meta-analysis, the lifetime prevalence of AUD in patients with schizophrenia is 24.3% [1]. Several theories account for the high rate of co-occurring substance use disorder in schizophrenia [2, 3]. The “two-hit” model argues that combined genetic risk and alcohol drinking during adolescence contribute to the development of schizophrenia and AUD. The self-medication theory is that patients with schizophrenia tend to find substance use a relief method for psychiatric symptoms or side effects of antipsychotics [2]. The reward deficiency syndrome explains that a transient improvement of dysfunctional dopamine-mediated mesocorticolimbic brain-reward system may underlie the primary biological effect of substances abuse [4]. The neonatal ventral hippocampal lesion (NVHL) model, which was led by the neurodevelopmental theory of schizophrenia, also has been introduced to explain the high prevalence of AUD among patients with schizophrenia [2]. In this animal model of schizophrenia, rats who received small, bilateral hippocampal lesions at the end of the first week of life manifest schizophrenia-like social deficits in adulthood [5]. A previous research showed that NVHL rats tend to consume more alcohol than the control group when they become adults after access to alcohol during adolescence [6].

Although there have been some prospective studies regarding moderate drinkers showing a U-shaped or sometimes J-shaped relationship between mortality rate and the amounts of alcohol consumption [7, 8], the comorbidity of AUD is associated with poor clinical outcomes of schizophrenia, which are low treatment compliance, an increased rehospitalization rate, and a high relapse rate [9, 10]. Schizophrenia patients with comorbid AUD experience adverse life events, such as unemployment and divorce [11]. In addition, the comorbid AUD negatively impacts patients with schizophrenia through poor adjustment toward lives and its association with depressive symptoms and disruptive behaviors [12]. A previous study reported that schizophrenia patients with AUD have more than a twofold risk of violence compared to those without AUD [13].

As aforementioned, previous studies on the effect of alcohol use on patients with schizophrenia showed that comorbid AUD aggravates psychiatric symptoms and treatment noncompliance. However, most previous studies had been conducted before the 2000s and had small sample size which was a total of less than 300 subjects. Considering that most randomized control trials recruit subjects from a few hospitals or medical institutions, the participants in previous studies could not have reflected the characteristics of the entire population of interest. Furthermore, many previous studies adopted a cross-sectional design, which was an obstacle in terms of examining the long-term effect of alcohol use [14].

The aim of the present study was to investigate clinical indicators of treatment, including the rates of psychiatric hospitalizations and emergency room (ER) visits, and medication possession ratio (MPR) in schizophrenia patients with comorbid AUD. MPR has been used for measuring medication adherence, which is calculated as a ratio of days of medication supply divided by the observation period. In order to overcome the limitations of previous studies, we used nationwide population data reflecting real-clinical practice in a large population. We analyzed these clinical outcomes in schizophrenia patients with and without comorbid AUD and time-varying patterns of clinical outcomes before and after the time point of diagnosis of AUD. We aimed to identify the effect of comorbidity of AUD on clinical outcomes of schizophrenia.

## Methods

### Data source

South Korea has a public medical insurance system based on fee-for-service, known as the National Health Insurance (NHI) system, covering approximately 98% of the country’s population [15, 16]. It is mandatory for all South Koreans to register with the NHI. Thus, medical claims of the entire population are recorded in the Health Insurance Review Agency (HIRA) database [17]. The claim data in the HIRA database includes information on patients’ visits or admissions to medical institutions, diagnoses, and demographics, such as age and sex [16, 18].

### Study sample and design

We used the HIRA claim data from January 1, 2007 to December 31, 2016. The following inclusion criteria were applied to identify an incident cohort of schizophrenia patients: (1) at least one claim of the diagnostic code F20 (schizophrenia) from January 1, 2008 to December 31, 2016 [The diagnostic code is based on the Korean Standard Classification of Diseases (KCD), a version of International Classification of Disease 10]; (2) no prescription of antipsychotic medication within 1 year preceding the diagnosis of schizophrenia; (3) starting of antipsychotic treatment within 3 days after the diagnosis of schizophrenia; and (4) onset age ranging from 12 to 80 years at the diagnosis of schizophrenia. The exclusion diagnostic criteria were psychotic disorder due to another medical condition, dementia, substance-induced psychotic disorder, substance intoxication with perceptual disturbances, moderate or severe intellectual disability, and autism spectrum disorder, which should not have been diagnosed before the diagnosis of schizophrenia.

Next, we selected the patients with comorbid AUD from the schizophrenia patients derived from the above inclusion and exclusion criteria. The comorbid AUD group was identified by the diagnostic code F10 (AUD) after the diagnosis of schizophrenia. The criteria listed in the Diagnostic and Statistical Manual of Mental Disorders (DSM)-5 was not used to diagnose AUD in this study because the only available information in the HIRA database regarding the diagnoses was based on the KCD codes. However, in South Korea, most psychiatrists in clinical practice have been trained to diagnose psychiatric disorders using DSM criteria, and the relevant KCD codes are only used for reimbursement. To evaluate the impact of the diagnosis of AUD on clinical outcomes of schizophrenia, we excluded patients who had been diagnosed schizophrenia and AUD simultaneously. Subsequently, we selected control patients with schizophrenia, matched for the onset age, sex, and observation period. We performed a matched case–control study, comparing the rates of psychiatric admissions and ER visits, and MPR between the comorbid AUD and control groups. Within-group analysis was also conducted to investigate differences in clinical outcomes before and after the time point of diagnosis of AUD within each group.

This study was approved by the Institutional Review Board (IRB) of the Asan Medical Center (IRB No. 2018-0131). The requirement for informed consent was exempted because of the use of anonymous and de-identified data.

### Definitions of outcomes

Main outcomes included the rates of psychiatric admissions and ER visits, and MPR. Psychiatric admission and ER visit have been used as the outcome reflecting the prognosis of several psychiatric disorders in previous studies [6, 14, 19]. Psychiatric admission was determined as hospitalization of which the main diagnosis was a psychiatric disorder. The duration of admission ranged from 14 to 180 days. We removed the events of hospitalization that had occurred within 30 days from the prior discharge. MPR is defined as the proportion of days of medication supply during a specified time period. Increased MPR indicates a high level of medication adherence, whereas decreased MPR indicates the opposite [20]. In this study, MPR was calculated as a ratio of days of medication supply divided by the follow-up period.

We also calculated the number of treatment discontinuations and duration of the first antipsychotic treatment episode. Treatment discontinuation was defined as no antipsychotic prescription within 28 days from the expected date of the next antipsychotic prescription. The first antipsychotic treatment episode was defined from the date of the schizophrenia diagnosis to the first treatment discontinuation.

### Statistical analyses

We compared continuous and categorical variables using Student’s *t* and Chi-square tests, respectively. Multivariate linear regression analysis was performed for the interaction effect of dependent variables between comorbid AUD and control groups. For post hoc tests, the unpaired *t*-test was used for between-group analyses and paired *t*-test for within-group analyses. The statistical significance level was set at 0.05. All data were processed using the R program ver. 3.5.1 (R Development Core Team, Vienna, Austria).

## Results

### Demographic and clinical characteristics of the study population

Of a total of 64,442 patients with incident first-episode schizophrenia, 1598 (2.48%) patients were included in the comorbid AUD group. There were significant differences in the onset age, sex, and observation period between schizophrenia with and without AUD groups (*n* = 62,844). The comorbid AUD group showed a higher onset age (44.3 ± 12.2 vs. 40.8 ± 15.7 years, *p* < 0.001), greater male prevalence (75.1% vs. 44.8%, *p* < 0.001), and longer observation period (5.1 ± 2.5 vs. 4.0 ± 2.8 years, *p* < 0.001) compared to the control group. Of the 62,844 schizophrenia patients without AUD, 1598 patients were selected as the controls which were matched for the onset age, sex, and observation period (Table 1).

**Table 1:** Demographic and clinical characteristics of the study population

Variable	Schizophrenia with AUD (*n* = 1598)	Matched controls (*n* = 1598)	*p*-value
Onset age for schizophrenia, mean (SD), years	44.3 (12.2)	44.2 (12.4)	0.873
Onset age for comorbid AUD, mean (SD), years	46.1 (12.1)	NA	
Sex, *n* (%)			1.000
Male	1200 (75.1)	1199 (75.0)	
Female	398 (24.9)	399 (25.0)	
Observation period, mean (SD), years	5.1 (2.5)	5.1 (2.5)	0.941
Total duration of antipsychotic treatment, mean (SD), years	2.6 (2.2)	3.5 (2.5)	< 0.001
Duration of the first antipsychotic treatment, mean (SD), days	202.6 (337.8)	344.0 (507.9)	< 0.001
Number of antipsychotic drugs in the total observation period, mean (SD)	4.0 (2.0)	3.4 (1.9)	< 0.001
Number of the treatment discontinuations^a^ in the total observation period, mean (SD)	3.4 (3.0)	2.6 (2.9)	< 0.001

*AUD* alcohol use disorder, *SD* standard deviation, *NA* not applicable

^a^Defined as no antipsychotic prescription within 28 days from the expected date of the next prescription

Compared to the control group, the comorbid AUD group was prescribed a higher number of antipsychotics during the total observation period (4.0 ± 2.0 vs. 3.4 ± 1.9, *p* < 0.001). The mean total duration of antipsychotic treatment was significantly longer in the control group than in the comorbid AUD group (3.5 ± 2.5 vs. 2.6 ± 2.2 years, *p* < 0.001). There were significant differences in the mean number of treatment discontinuations and duration of the first antipsychotic treatment between the two groups. On average, the comorbid AUD and control groups had 3.4 ± 3.0 and 2.6 ± 2.9 treatment discontinuations, respectively. The mean duration of the first antipsychotic treatment was significantly longer in the control group than in the comorbid AUD group (344.0 ± 507.9 vs. 202.6 ± 337.8 days, *p* < 0.001).

### Main outcomes

**Fig. 1:**
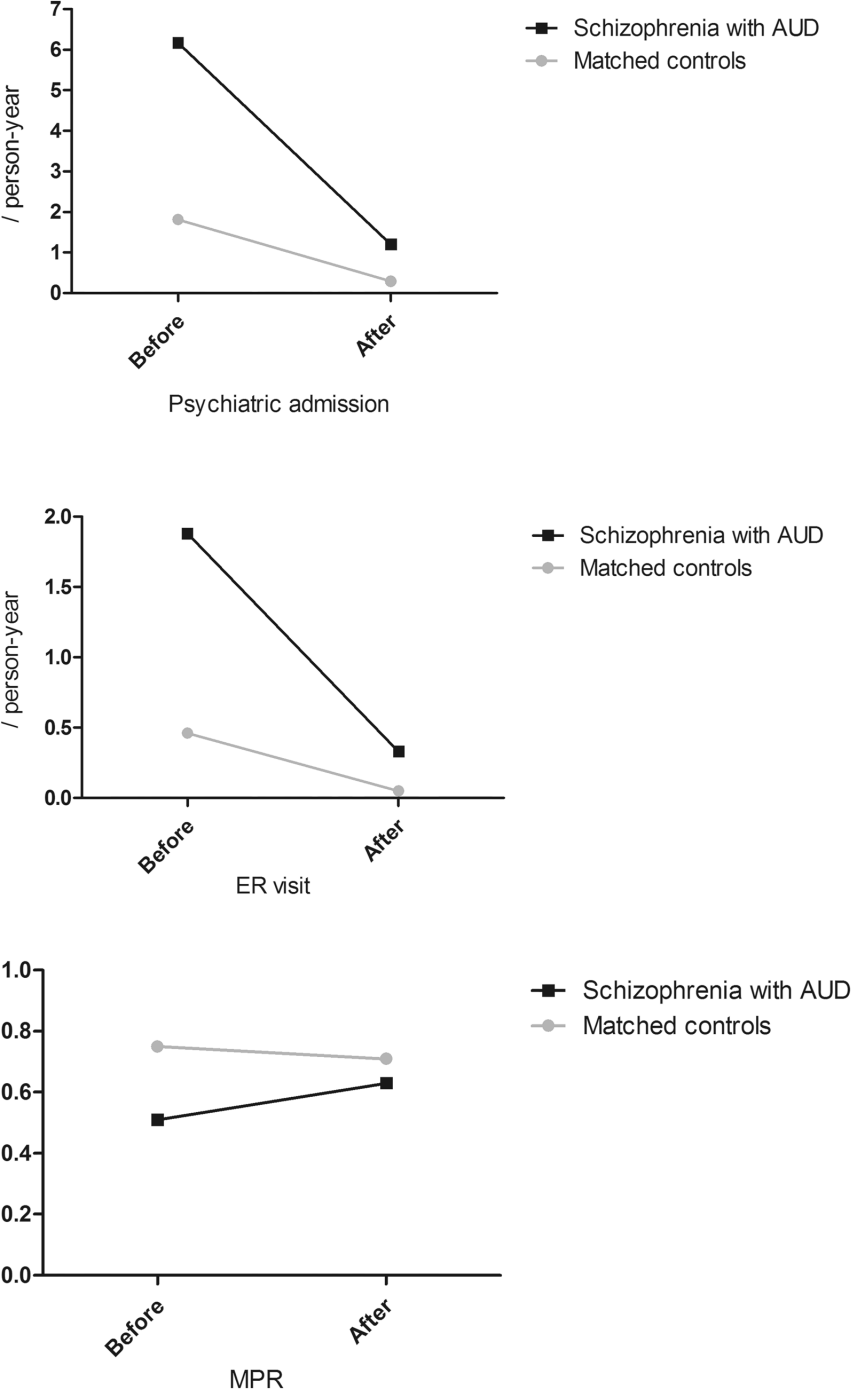
The rates of psychiatric admissions and ER visits, and MPR among schizophrenia patients with AUD and matched controls. *AUD* alcohol use disorder, *ER* emergency room, *MPR* medication possession ratio

The decrease in the rates of psychiatric admissions and ER visits was greater in the comorbid AUD group compared to the control group, with significant group-by-time interaction effects (psychiatric admission: *t* = − 4.604, *p* < 0.001; ER visit: *t* = − 2.456, *p* = 0.014). While the control group showed a decreasing MPR over time, the comorbid AUD group had an increased MPR after the diagnosis of AUD with a significant group-by-time interaction effect (*t* = 9.180, *p* < 0.001) (Fig. 1).

Table 2 exhibits the results of both between- and within-group analyses. All outcome variables were worse in the comorbid AUD group compared to the control group both before and after the diagnosis of AUD. Higher rates of psychiatric admissions (*t* = 5.826, *p* < 0.001) and ER visits (*t* = 3.074, *p* = 0.002) and lower MPR (*t* = 16.346, *p* < 0.001) were found in the comorbid AUD group than in the control group before the diagnosis of AUD.

**Table 2:** Within- and between-group comparisons in the rates of psychiatric admissions and ER visits, and MPR

Variable	Schizophrenia with AUD (*n* = 1598)			Matched controls (*n* = 1598)			Between-group *t*-value^a^		Interaction effect (group * time) *p*-value^b^
	Before	After	Within-group *t*-value^a^	Before	After	Within-group *t*-value^a^	Before	After	
Psychiatric admission, /person-year, mean (SD)	6.17 (29.63)	1.20 (2.35)	6.684***	1.81 (4.14)	0.29 (1.12)	14.168***	5.826***	13.974***	< 0.001
ER visit, /person-year, mean (SD)	1.88 (18.32)	0.33 (1.06)	3.377***	0.46 (2.30)	0.05 (0.31)	7.062***	3.074**	10.135***	0.014
MPR, mean (SD)	0.51 (0.41)	0.63 (0.31)	9.333***	0.75 (0.42)	0.71 (0.28)	3.168**	16.346***	7.656***	< 0.001

*AUD* alcohol use disorder, *SD* standard deviation, *ER* emergency room, *MPR* medication possession ratio

^a^Unpaired *t*-test was used for between-group analyses and paired *t*-test for within-group analyses

^b^Multivariate linear regression analysis was used for the interaction effect

**p = 0.002

***p < 0.001

In the comorbid AUD group, the rates of psychiatric hospitalizations (*t* = 6.684, *p* < 0.001) and ER visits (*t* = 3.377, *p* < 0.001) significantly decreased after the diagnosis of AUD, and MPR significantly increased after the diagnosis of AUD (*t* = 9.333, *p* < 0.001). In the control group, after the time point of diagnosis of AUD, the rate of psychiatric admissions (*t* = 14.168, *p* < 0.001) and ER visits (*t* = 7.062, *p* < 0.001), and MPR (*t* = 3.168, *p* = 0.002) significantly decreased.

## Discussion

To the best of our knowledge, this is the first nationwide population study regarding the effect of alcohol use on clinical outcomes of schizophrenia. The HIRA claim database was used to include the incident cohort of schizophrenia, from which the comorbid AUD group was selected as the case group. With matched controls, between- and within-group analyses were conducted to compare the rates of psychiatric admissions and ER visits, and MPR. The comorbid AUD group had significantly higher rates of psychiatric admissions and ER visits and lower MPR, compared to the control group before and after the diagnosis of AUD. Significantly greater decrease in the rates of psychiatric admissions and ER visits was observed in the comorbid AUD group after the diagnosis of AUD, compared to the control group. Contrary to a decreased MPR after the time point of diagnosis of AUD in the control group, MPR increased after the diagnosis of AUD in the case group.

In our study, all outcome variables were worse in the comorbid AUD group compared to the control group both before and after the time point of diagnosis of AUD. Consistent with the current findings, several previous studies have reported the association of alcohol use with poor clinical outcomes in schizophrenia. Drake et al. examined the pattern of alcohol use among 115 patients with schizophrenia and found the association of alcohol use with medication noncompliance, increased symptomatology, and a higher rate of rehospitalization, emphasizing that even minimal alcohol use is closely related to rehospitalization [21]. The poor clinical outcomes in the comorbid AUD group might be related to severe psychiatric symptom severity. A previous study by Margolese et al., which was a cross-sectional survey to document substance abuse in 207 schizophrenia and related psychoses patients, found that current dual diagnosis patients had significantly higher Positive and Negative Syndrome Scale scores than single diagnosis patients [14]. Notably, we found significant improvement in all outcome variables after the diagnosis of AUD in the comorbid AUD group, with significant group-by-time interaction effects. Based on previous studies [9, 10] reporting poor clinical outcomes in schizophrenia associated with comorbid AUD, we anticipated that changes in the clinical outcomes before and after the diagnosis of AUD in the comorbid AUD *group would result in poor prognosis.* However, the findings contradicted our anticipation. The significant improvement after the diagnosis of AUD could be attributed to clinicians’ reconsidering other factors impacting clinical outcomes, such as family education, a psychiatric history, and engagement in psychiatric care in the community when making the comorbid diagnosis of AUD.

To date, there has been no well-designed comparative study about antipsychotic treatment between schizophrenia patients with and those without comorbid AUD. We observed that the comorbid AUD group had a shorter mean total duration of antipsychotic treatment and higher number of treatment discontinuations in the total observation period, in comparison with the control group. Along with a higher mean number of antipsychotics in the total observation period in the case group than in the control group, the current results may indicate that antipsychotic treatment in patients with comorbid AUD would be challenging, given the possibility of a lower treatment compliance and higher number of antipsychotics to control psychiatric symptoms in the comorbid AUD group derived from the current findings. We investigated the pattern of antipsychotic treatment in the comorbid AUD group in an exploratory manner, therefore further details on antipsychotic treatment in schizophrenia patients with comorbid AUD should be investigated in future studies.

Although the sample size of the current study was sufficiently large, the prevalence of AUD among schizophrenia patients was lower than that in previous studies. A higher prevalence of alcohol abuse in patients with psychotic disorders has been reported, in comparison with the general population [2, 22]. Meta-analyses have reported a median prevalence of current AUD of 9% and a lifetime prevalence of AUD of 24.3% in schizophrenia patients [1, 11]. The discrepancy from previous literature may be attributed to the use of claim data derived from clinical practice in this study. Considering that clinicians tend to focus on chief complaints, which are mainly auditory hallucinations or delusions in the case of patients with a psychotic disorder, comorbid psychiatric diagnoses could be neglected in clinical practice, especially considering the very low prevalence of illegal drug abuse and dependence in South Korea [23]. A mean observation period of 5.1 years in this study might not be sufficiently long to determine the lifetime prevalence of AUD among patients with schizophrenia. In this study, the onset age and sex ratio differed between the comorbid AUD and control groups. Compared with the control group, the case group had a higher mean onset age of schizophrenia and longer mean duration of observation period. Significantly higher male ratio was found in the comorbid AUD group than in the control group. These findings might indicate the distinct characteristics of schizophrenia patients with comorbid AUD from those without comorbid AUD. The late-onset age of schizophrenia in the patients with comorbid AUD [24, 25] or a history of substance abuse [26] has been reported in previous studies. The larger proportion of male in the comorbid AUD group in this study was also in accordance with the results from previous studies on AUD in schizophrenia [9, 24, 26, 27].

Previous studies have shown the high prevalence and risk of substance abuse among individuals with psychotic disorders [2, 9, 22]. However, most of the existing literature on alcohol abuse in schizophrenia patients had been conducted in the 1990s and had several disadvantages including small sample size, a cross-sectional design, or a short observation period. Our study had large sample size and utilized various types of variables as the marker of clinical outcomes. In addition to between-group comparisons, we also performed within-group comparisons to observe the time-varying patterns of dependent variables. This noble comparative design has not been adopted in previous studies on AUD in schizophrenia.

This study had a few limitations. First, it was based on the claim data from clinical practice, not created for research purposes, introducing a possibility of disregarding comorbid psychiatric diagnoses. Second, the claim data could not provide information regarding additional characteristics of schizophrenia, such as severity of positive or negative symptoms, what support network the schizophrenia patients had, and whether there exist remissions or partial remissions during the observation period or not. This should be considered in interpreting the results. Third, the patients with comorbid AUD in this study might not be representative of the entire population of interest. The comorbid AUD group could be composed of severe cases of AUD, given the high prevalence of alcohol-related disorders in South Korea [28, 29], which suggests that mild alcohol abuse could be neglected in clinical practice. Fourth, even though we used nationwide population data, the generalizability of our results might be limited due to the low prevalence of illegal drug use and dependence in South Korea South [23]. Finally, we could not obtain information on the treatment for AUD in the comorbid AUD group. Whether the schizophrenia patients with comorbid AUD had been treated regularly or not could affect the clinical course of schizophrenia. It is worth performing further analyses for comparing clinical outcomes in the comorbid AUD group according to their treatment compliance for AUD.

The diagnosis of comorbid AUD can put a considerable effect on overall clinical outcomes of schizophrenia. Clinical outcomes in the comorbid AUD group were worse even before the diagnosis of AUD. This implies that clinicians should consider the possibility of alcohol abuse in the case of schizophrenia patients with poor treatment outcomes within the first several years after the diagnosis of schizophrenia. The current findings need to be verified by further research to achieve confirmative evidence.

## Conclusions

Having a comorbid AUD can put a substantial impact on overall clinical outcomes of first-episode schizophrenia. There were significantly higher rates of psychiatric admissions and ER visits and lower MPR in the comorbid AUD group before and after the diagnosis of AUD, compared to the control group. After the diagnosis of AUD, clinical outcomes significantly improved in the comorbid AUD group. These findings emphasize that comorbid AUD in schizophrenia patients should be monitored carefully. Clinicians should recognize psychiatric comorbidities even when they evaluate patients with schizophrenia in early-phase. Further research is required to provide confirmatory findings of the association of AUD with schizophrenia.

## Data Availability

Data availability is not applicable to this article as no new data were created or analyzed in this study.

## Abbreviations

AUD: Alcohol use disorder
ER: Emergency room
MPR: Medication possession ratio
NHI: National Health Insurance
HIRA: Health Insurance Review Agency
KCD: Korean Standard Classification of Diseases
ICD-10: International Classification of Disease 10
IRB: Institutional Review Board
